# Molecular characterization of swine acute diarrhea syndrome coronavirus detected in Vietnamese pigs

**DOI:** 10.1186/s13567-024-01445-0

**Published:** 2025-01-09

**Authors:** Nam Phuong Le, Bac Tran Le, Van Phan Le, Jung-Eun Park

**Affiliations:** 1 College of Veterinary Medicine, Chungnam National University, Daejeon, Republic of Korea; 2 College of Veterinary Medicine, Vietnam National University of Agriculture, Hanoi, Vietnam

**Keywords:** Swine acute diarrhea syndrome coronavirus, spike, pig, Vietnam

## Abstract

Swine acute diarrhea syndrome coronavirus (SADS-CoV) is a novel porcine coronavirus that was recently identified in southern China in 2017. At present, there is a lack of nationwide epidemiological investigations of the newly emerged SADS-CoV. Because Vietnam is geographically adjacent to southern China, many diseases have spread from China to Vietnam. To assess SADS-CoV transmission to Vietnam, we conducted a retrospective study to detect SADS-CoV in samples collected from pig farms in northern Vietnam. Among the 69 fecal samples tested, 5 were positive for SADS-CoV. The spike gene sequence showed high genetic homology with strains circulating in China. Our study is the first to show that SADS-CoV has spread from China to Vietnam and highlights the need for global epidemiological investigations of SADS-CoV.

**Supplementary Information:**

The online version contains supplementary material available at 10.1186/s13567-024-01445-0.

## Introduction, methods and results

Coronaviruses (CoVs), members of the *Coronaviridae* family, are a group of single-stranded positive-sense, enveloped RNA viruses that cause respiratory and intestinal diseases in diverse hosts, including birds, mammals, and humans [1]. In the twenty-first century, three zoonotic CoVs emerged and spread globally, posing severe threats to public health and the economy [2–4]. To date, six CoVs have been identified to infect pigs, including four alpha-CoVs, transmissible gastroenteritis virus (TGEV), porcine epidemic diarrhea virus (PEDV), swine acute diarrhea syndrome coronavirus (SADS-CoV), and porcine respiratory coronavirus (PRCV); one beta-CoV, porcine hemagglutinating encephalomyelitis virus (PHEV); and one porcine delta-coronavirus (PDCoV) [5, 6]. Among these viruses, TGEV, PEDV, PDCoV, and SADS-CoV cause enteric coronavirus disease, which is characterized by acute diarrhea leading to high mortality in neonatal piglets or moderate mortality in sows and significant economic losses for the pork industry worldwide [7, 8].

SADS-CoV, also known as swine enteric alphacoronavirus (SeACoV) or porcine enteric alphacoronavirus (PEAV), is a novel porcine CoV first identified in southern China in 2017 [9–11]. SADS-CoV causes swine enteric diseases characterized by watery diarrhea and weight loss [10–12]. Coinfection with PEDV, PDCoV and SADS-CoV in swine has been reported, and the clinical manifestations and pathological features of these viral infections are extremely similar [13]. The SADS-CoV viral genome is closely related to the *rhinolophus* bat coronavirus HKU2, suggesting that this virus originated in bats [10, 11]. The virus can replicate in many cell types, including human cells [14, 15]. This raises concerns about the complexity of disease control and the possibility of zoonotic transmission.

At present, there is a lack of nationwide epidemiological investigations of the newly emerged SADS-CoV. Therefore, comprehensive and systematic research is urgently needed. Because Vietnam is geographically adjacent to southern China, many diseases have spread from China to Vietnam or vice versa. To assess SADS-CoV transmission to Vietnam, we conducted a retrospective study to detect SADS-CoV in samples collected from pig farms in northern Vietnam.

From 2018 to 2023, a total of 69 fecal samples in isolation medium were sent to the Faculty of Veterinary Medicine, Vietnam National University of Agriculture, from porcine diarrhea outbreaks in pig herds in northern and north-central provinces in Vietnam for the detection of porcine enteric CoV agents, including PEDV, PDCoV, and SADS-CoV. The samples were subjected to extraction of DNA/RNA genomic materials for molecular detection and genomic sequencing using the MagMAX Pathogen RNA/DNA Kit (Applied Biosystems) following the manufacturer’s instructions. Multiplex RT‒qPCR was used to detect PEDV, PDCoV, and SADS-CoV following our previous development kit [16]. Among the 69 fecal samples obtained from piglets with diarrhea, 5 were positive for SADS-CoV, with quantification cycle values ranging from 32.80 to 36.17. The detailed information and geographic distribution of the SADS-CoV-positive samples are summarized in Table 1. The years in which these samples were collected varied; one was from 2019, one was from 2021, and three were from 2023. The locations where these samples were collected also varied; one was from Bac Ninh, one was from Ha Noi, and three were from one swine farm in Thanh Hoa. Three fecal samples from Thanh Hoa were coinfected with PEDV, and two fecal samples from Bac Ninh and Ha Noi were coinfected with PEDV and PDCoV.

**Table 1:** **Information on SADS-CoV-positive fecal samples**

Name	Sample	Collection date	District	Province	Positivity
VNUA-BN01/2019	Feces	2019.01.08	–	Bac Ninh	SADS-CoV, PEDV, PDCoV
VNUA-HN01/2021	Feces	2021.03.12	Ha Tay	Ha Noi	SADS-CoV, PEDV, PDCoV
VNUA-TH01/2023	Feces	2023.10.18	Thach Thanh	Thanh Hoa	SADS-CoV, PEDV
VNUA-TH02/2023	Feces	2023.10.18	Thach Thanh	Thanh Hoa	SADS-CoV, PEDV
VNUA-TH03/2023	Feces	2023.10.18	Thach Thanh	Thanh Hoa	SADS-CoV, PEDV

The positive samples containing SADS-CoV were subjected to viral genome sequencing targeting the spike (S) gene. Viral RNA was extracted using the MagMAX Pathogen RNA/DNA Kit (Applied Biosystems). cDNAs were synthesized using the iScript™ cDNA Synthesis Kit (Bio-Rad). Nested PCR was performed to target the S gene using Q5 high-fidelity DNA polymerase (NEB). The primers used for nested PCR are listed in Additional file 1. The PCR mixture contained 10 μL of 5 × Q5 reaction buffer, 1 μL of dNTPs (10 nM), 0.5 μL of Q5 High-Fidelity DNA Polymerase (2 U/μL), 1 μL of inner or outer primer pairs (10 pmol), 3 μL of cDNA for the first round or DNA of the first round of PCR product for the second round of template, and a final water reaction volume of 50 μL. The PCR thermocycling procedure was performed at 98 °C for 20 s, 52 °C in the first round or 54 °C in the second round for 30 s, and 72 °C for 120 s for 35 cycles. The PCR products were purified and subjected to direct sequencing via Sanger sequencing (Cosmogenetech, South Korea). The full and partial S gene was successfully amplified from four of the five positive samples. A total of 3793 nucleotides of the S gene sequence were submitted to GenBank under accession numbers PP062883, PP062884, and PP062886. A total of 1578 nucleotides of the S gene sequence were submitted to GenBank under accession number PP062885. The sequence information for all four SADS-CoVs was identical.

**Figure 1:**
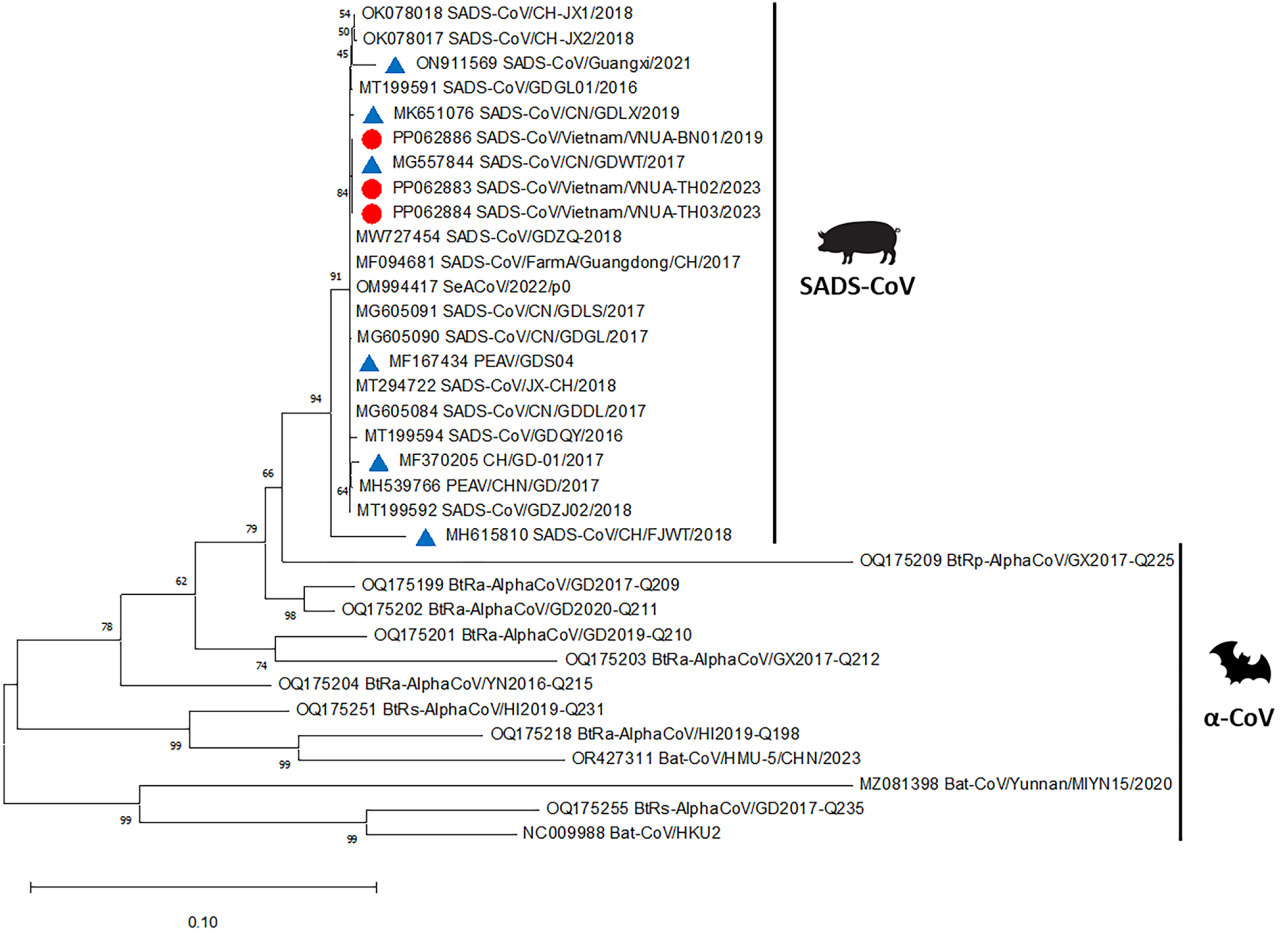
**Phylogenetic analysis of SADS-CoV S genes detected in Vietnam.** The red circles indicate the samples from this study. The blue triangles indicate the reference strains. GenBank accession numbers are provided for all the sequences. Scale bars indicate nucleotide substitutions per site.

**Figure 2:**
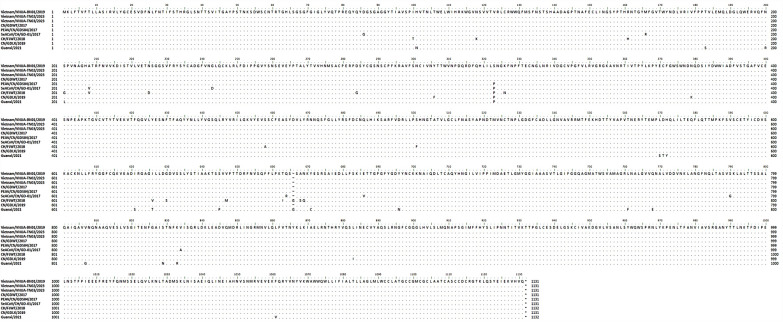
**Multiple sequence alignment of the SADS-CoV S protein.** Conserved amino acids are indicated with a dot (.) and deleted/added amino acids are indicated with a tilde ( ~). The alignment was performed using Clustal Omega.

The full S sequences of Vietnamese isolates and 31 reference strains, including SADS-CoV and bat-related SADS-CoV, were used for analysis. Phylogenetic analysis of the S gene sequences was conducted using Molecular Evolutionary Genetics Analysis (MEGA) software (version 11.0). Evolutionary distances were calculated via the neighbor‒joining method. Bootstrap iterations (*n* = 1000) with statistical values above 60% are presented to indicate reliability. Phylogenetic analysis revealed that these viruses were closely related to the SADS-CoV strains that circulated in China from 2017 to 2018 (Figure 1). The nucleotide and amino acid sequences were aligned through multiple sequence alignment using the ClustalW algorithm in BioEdit software (version 7.0). The Vietnamese SADS-CoV S strains had 100% nucleotide and 100% amino acid homology to the SADS-CoV/CN/GDWT/2017 strain (Table 2 and Figure 2). The Vietnamese SADS-CoV S strains had 99.9% nucleotide and 99.9% amino acid (single change at position 322, S to P) homology to the PEAV/GDS04 strain (Table 2 and Figure 2). These strains presented relatively low similarity with the CH/FJWT/2018 or Guangxi/2021 strains.

**Table 2:** **Homology of the nucleotide and deduced amino acid sequences of the full SADS-CoV S protein of the Vietnam isolates and the reference strains**

Strain	Vietnam/VNUA-BN01/2019		Vietnam/VNUA-TN02/2023		Vietnam/VNUA-TN03/2023	
	Nucleotide (%)	Amino acid (%)	Nucleotide (%)	Amino acid (%)	Nucleotide (%)	Amino acid (%)
Vietnam/VNUA-BN01/2019	–					
Vietnam/VNUA-TN02/2023	**100**	*100*	–			
Vietnam/VNUA-TN03/2023	**100**	*100*	**100**	*100*	–	
CN/GDWT/2017	**100**	*100*	**100**	*100*	**100**	*100*
PEAV/CN/GD-01–2017	**99.9**	*99.9*	**99.9**	*99.9*	**99.9**	*99.9*
SeACoV/CH/GD-01/2017	**99.5**	*99.2*	**99.5**	*99.2*	**99.5**	*99.2*
CH/FJWT/2018	**97.3**	*98.4*	**97.3**	*98.4*	**97.3**	*98.4*
CN/GDLX/2019	**99.8**	*99.6*	**99.8**	*99.6*	**99.8**	*99.6*
Guanxi/2021	**99.0**	*98.2*	**99.0**	*98.2*	**99.0**	*98.2*

Nucleotide identity (%) is shown in bold, and deduced amino acid identity (%) is shown in *italics.*

## Discussion

SADS-CoV was first detected in Guangdong Province, China, in 2017. During early outbreaks, the SADS-CoV genome sequences documented from three independent groups were 99.8–100% identical to each other. These include PEAV GDS04 [9], SeACoV CH/GD-01/2017 [10], and SADS-CoV CN/GDWT/2017 [11]. A retrospective investigation of SADS-CoV at 45 pig farms in Guangdong Province revealed that SADS-CoV emerged in Guangdong Province as early as August 2016 [13]. From May 2017 to January 2019, no new SADS-CoV cases arose in Guangdong pig herds. However, in 2018, a new SADS-CoV/CH/FJWT/2018 was identified in pigs in Fujian Province, which neighbors Guangdong Province [17]. In addition, SADS-CoV/CN/GDLX/2019 reemerged in pig herds in Guangdong starting in February 2019 [18]. Most recently, in May 2021, SADS-CoV/Guangxi/2021 was identified in Guangxi Province [19]. From 2021 to 2023, Zhang et al. conducted surveillance on pig farms in central China for porcine coronaviruses and confirmed that SADS-CoV was the cause of death in 400 piglets in Henan Province, Central China [20]. The strain causing the outbreak was designated SADS-CoV/HNNY/2023. Prior to 2021, SADS-CoV was observed only in coastal China, but the 2023 outbreak in Henan Province strongly suggests that the virus may have spread widely to inland areas of China. In addition, the seroprevalence of SADS-CoV antibodies determined by indirect enzyme-linked immunosorbent assay has ranged from 59.97% to 81.7% [21, 22], suggesting that SADS-CoV has a substantially wider spread than expected in China. SADS-CoV has not been reported in any other region in the world thus far.

To detect SADS-CoV outbreaks in Vietnam, we investigated the presence of SADS-CoV in piglets from swine farms with watery diarrhea in Vietnam from 2018 to 2023. SADS-CoV was detected in 5 of the 69 fecal samples. The results revealed that the first SADS-CoV-positive sample was collected in January 2019 from a farm located in Bac Ninh, suggesting that SADS-CoV infection had occurred in Vietnamese pigs since at least January 2019. During the study period, the SADS-CoV positivity rate in Vietnamese pigs was 7.25%, which was lower than the positivity rates of 10.29% [23] or 43.53% [13] reported in southern China. Compared with the higher positivity rates reported in southern China, the lower rate detected here could be partly due to these study limitations rather than reflecting an actual difference in virus prevalence. Sequencing analysis revealed that the entire S sequences of the three SADS-CoVs were identical, suggesting that these three SADS-CoV sequences may have the same origin.

We examined the molecular characteristics of the detected viruses to determine how they were transmitted to Vietnam. Sequence alignment revealed that amino acid identities between Vietnamese SADS-CoV and reference SADS-CoV strains ranged from 99.3% to 100%. Among them, Vietnamese SADS-CoV shares 100% homology with SADS-CoV/CN/GDWT/2017. Since SADS-CoV was first reported in Guangdong, it has subsequently spread to the neighboring provinces Fujian, Jiangxi, Guangxi and Henan [17, 19, 20]. This study relied on a limited number of fecal samples collected from specific high-density livestock regions in northern Vietnam, primarily around Bac Ninh, which restricts the generalizability of the results. The Bac Ninh is geographically close to southern China, where SADS-CoV was identified as an outbreak. This limited sampling scope may not capture the full extent of SADS-CoV spread across Vietnam, but from our data, SADS-CoV was introduced into northern Vietnam from southern China and gradually spread to northcentral Vietnam. Additionally, given its high homology with the GDWT/2017 strain, it was most likely transmitted in the Guangdong region.

Similar to previous findings in China, SADS-CoV-positive pigs in Vietnam were coinfected with PEDV or PDCoV [13, 23, 24]. These coronaviruses have similar clinical symptoms, so differential diagnosis can be made only through laboratory methods. Because the clinical symptoms are similar, the introduction of SADS-CoV likely went undetected. PEDV is currently spreading worldwide, which may cause the introduction of SADS-CoV to be overlooked. Therefore, it is necessary to diagnose SADS-CoV infection through accurate differential diagnosis.

Our study has limitations in that it was conducted in a limited geographic area and used a limited number of stool samples. These limitations do not provide information on the chronology and timing of SADS-CoV introduction. Our results suggest that SADS-CoV infections began approximately 2019 but do not provide information on the exact timing of introduction. Furthermore, we cannot rule out the possibility that the virus was introduced from Vietnam into China. Expanding sampling to include all regions of Vietnam would help clarify the extent of cross-border transmission and identify potential hotspots of SADS-CoV. Future investigations that expand sampling and geographic analysis, especially in areas bordering China, are essential to understand cross-border transmission of the virus and to gain a more comprehensive view of the spread of SADS-CoV in Vietnam. These findings may also help in the detection of potential emerging areas of SADS-CoV and support targeted prevention and control efforts.

In conclusion, to our knowledge, this is the first report on the spread of SADS-CoV outside China. We detected SADS-CoV in piglets with diarrhea in Vietnam. Our study provides insights into the potential transmission of SADS-CoV between China and Vietnam, as well as the occurrence of the virus in other countries. However, how and when the virus was introduced into pig farms in Vietnam remain to be determined. Thus, further studies and continuous biosurveillance are needed to monitor SADS-CoV in pigs in Vietnam.

## Supplementary Information

**Additional file 1**.** Primer information used in the study.**

## Data Availability

The datasets used and analyzed in the study are available from the corresponding author on reasonable request.
