# Supplementary material for: Molecular characterization of swine acute diarrhea syndrome coronavirus detected in Vietnamese pigs
Source: Vet Res. 2025 Jan 9;56:4. doi: 10.1186/s13567-024-01445-0 (PMC11720870; doi:10.1186/s13567-024-01445-0)
Supplement: Supplementary file 1 — Additional file 1. Primer information used in the study. [file 13567_2024_1445_MOESM1_ESM.docx]

**Additional file 1. Primer information used in the study**

| **Name** | **Sequences (5’ to 3’)** | **Location** | **PCR product size (bp)** |
| --- | --- | --- | --- |
| SADS-S1-out-F | GGATAAAGACATTACTGATATGGTGC | -107 | 2064 |
| SADS-S1-out-R | CATTGAATCTATCAGTAGTGGGCA | 1957 |  |
| SADS-S1-in-F | GCAACCATCTTGTAACAACTAAATG | -21 | 1860 |
| SADS-S1-in-R | CAAACATAGCAATTCTGCCACGT | 1391 |  |
| SADS-S2-out-F | TGCAAACTACAATGCCTAAGTTCAG | 1736 | 1952 |
| SADS-S2-out-R | ACGGGACTAGTTTTGGCAGCAA | +215 |  |
| SADS-S2-in-F | AGTTGCGAGACTTACATATGTGA | 1765 | 1642 |
| SADS-S2-in-R | GTGTCAACTGGAAAAGTCCACCA | +26 |  |
